# Molecular Design of d-Luciferin-Based Bioluminescence and 1,2-Dioxetane-Based Chemiluminescence Substrates for Altered Output Wavelength and Detecting Various Molecules

**DOI:** 10.3390/molecules26061618

**Published:** 2021-03-15

**Authors:** Hideo Takakura

**Affiliations:** Laboratory of Bioanalysis and Molecular Imaging, Graduate School of Pharmaceutical Sciences, Hokkaido University, Sapporo, Hokkaido 060-0812, Japan; htakakura@pharm.hokudai.ac.jp

**Keywords:** bioluminescence, chemiluminescence, imaging, near infrared, molecular probes, design strategy

## Abstract

Optical imaging including fluorescence and luminescence is the most popular method for the in vivo imaging in mice. Luminescence imaging is considered to be superior to fluorescence imaging due to the lack of both autofluorescence and the scattering of excitation light. To date, various luciferin analogs and bioluminescence probes have been developed for deep tissue and molecular imaging. Recently, chemiluminescence probes have been developed based on a 1,2-dioxetane scaffold. In this review, the accumulated findings of numerous studies and the design strategies of bioluminescence and chemiluminescence imaging reagents are summarized.

## 1. Introduction

Luminescence imaging is a powerful method for detecting biomolecules and physiological phenomena, as well as monitoring gene expression in living animals [1,2]. Luminescence imaging is advantageous because, unlike fluorescence imaging, excitation light is not required; therefore, scattered light and tissue autofluorescence is not observed. In addition, background signals in luminescence imaging can be very low, allowing for highly sensitive measurements. To date, bioluminescence in vivo imaging has been used in almost all cases of luminescence imaging of living animals. However, recently developed chemiluminescent compounds with high brightness have a great potential for chemiluminescence in vivo imaging and may be used instead of bioluminescence in some cases in the future [3].

In order to detect signals from deeper tissues in animals, it is necessary to consider the wavelength of luminescence. Visible light in the region between 400 nm and 600 nm is attenuated due to absorption by hemoglobin and oxyhemoglobin in the blood [4]. Therefore, for in vivo imaging, it is desirable to use light in the near-infrared (NIR) region, which is less absorbed by hemoglobin and oxyhemoglobin, and is less scattering by tissue. Within the NIR region, the wavelength ranges of 650–900 nm and 1000–1700 nm are called NIR-I and NIR-II, respectively [5,6,7]. So far, bioluminescence imaging in the NIR-I region has been studied, and in recent years, the research on NIR-II region was also reported [8]. In addition, functionalized substrates, so called probes, have been developed to detect target biomolecules and biological events in living animals. Such functionalization has been accomplished by modification of luciferin based on rational molecular design and protein engineering of luciferase utilizing bioluminescence resonance energy transfer (BRET) and split luciferase complementation [1,9]. In this review, a summary is presented of the reported molecular design of substrates emitting NIR-I bioluminescence and probes with a focus on the firefly/click beetle luciferin-luciferase system. Similar studies have been carried out in a bioluminescent system using coelenterazine as a substrate, but refer to other excellent reviews [10,11]. There is also a bioluminescent system with bacterial luciferin-luciferase reaction, but it is not discussed in this review because it does not require the addition of luciferin from outside; therefore, there is no research on the molecular design of bacterial luciferin [12,13]. In this review, further, recent reports on chemiluminescent compounds and chemiluminescence probes are also briefly summarized.

## 2. Firefly/Click Beetle Bioluminescence

**Scheme 1:**
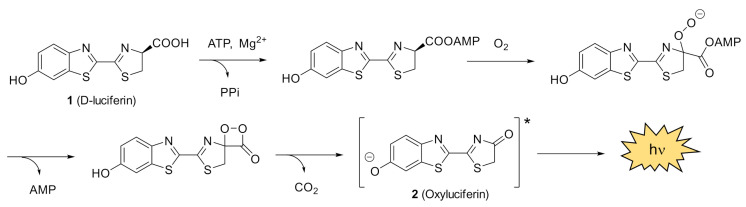
The chemical reaction of firefly/click beetle bioluminescence. In the reaction, d-luciferin is adenylated by luciferase using adenosine triphosphate (ATP) followed by reaction with molecular oxygen to generate a high-energy dioxetane intermediate. Along with the decomposition of the intermediate, oxyluciferin is excited and emits light.

The most common bioluminescent systems for in vivo imaging are the firefly/click beetle luciferin-luciferase reactions (Scheme 1). In this reaction, luciferase catalyzes the oxidation of d-luciferin (Scheme 1, Compound **1**) using oxygen molecules in the presence of adenosine triphosphate (ATP). First, ATP is used to form an adenylated luciferin, which then reacts with molecular oxygen to produce an excited state of a high energy dioxetane intermediate, oxyluciferin (Scheme 1, Compound **2**). Then, the excited oxyluciferin emits light. Although various firefly/beetle luciferases are known, all luciferases consume the same d-luciferin as a substrate. The luminescence wavelength depends upon the measurement conditions and the luciferase employed. Firefly luciferase from North America results in luminescence wavelengths of 558 nm in neutral to weakly alkaline conditions, but 610 nm in weakly acidic conditions [14]. The luminescence wavelength is also affected by temperature, with higher temperatures resulting in red-shifted luminescence wavelengths [15]. Other species of luciferases, such as Brazilian luciferase and Caribbean luciferase, result in luminescence wavelengths of 538 nm and 613 nm, respectively [16]. Interestingly, the luminescence wavelengths of these two luciferases are pH-independent. Other than these variants, many mutants with altered luminescence wavelengths are known, and most of them have a longer wavelength shift than that of wild type luciferase [17,18,19,20,21]

## 3. Bioluminescent System with Longer Wavelength

### 3.1. Molecular Mechanism of Bioluminescence Color

The molecular mechanism of the change in luminescence wavelength of firefly luciferin-luciferase is still under debate due to the complex chemical and photochemical properties of oxyluciferin, the bioluminescent emitter [22,23,24,25]. For example, oxyluciferin can exist in many different tautomeric and deprotonated forms, including the keto-enol-enolate and phenol-phenolate species. In addition, the p*K*a values of the keto and phenol groups and thought to be close to each other [25]. Moreover, the photochemical properties of each structure are very sensitive to the environment [22,23,24]. It is now believed that the structure of the active site emitter is oxyluciferin in its keto-phenolate form and that the change in emission wavelength is due to non-specific polarity, size of the active site, and specific electrostatic interactions. According to the results of studies on the photochemical properties of oxyluciferin, the maximum emission wavelength of oxyluciferin is about 640 nm. The currently reported variant of luciferase showing the longest wavelength is 622 nm in the railroad worm and 626 nm in the mutant, which is roughly consistent with the maximum wavelength [16,21]. However, these wavelengths fall short of the optical window. As long as d-luciferin is used as a substrate, it will be difficult to find variants or mutants with longer luminescence wavelengths.

### 3.2. Modification of Luciferase

In order to develop a bioluminescent system with luminescence wavelengths in the optical window, the modification of luciferase with fluorescent dyes that emit NIR-I light, such as AlexaFluor690 or AlexaFluor750, has been attempted [26]. However, since luciferase is chemically conjugated with the fluorescent dyes outside the cell, it is difficult to introduce them into the cell after conjugation. Another approach is to create a fusion protein that combines a fluorescent protein that emits NIR-I light with a luciferase via protein engineering [27]. In this case, it is possible to introduce the gene into the cell, making it possible to apply the fusion protein to culture cells or animals. To date, there have been reports on the development of fusion proteins that are composed of red-emitting fluorescent proteins and firefly luciferase; however, there have been no reports using NIR-I-emitting fluorescent proteins [28,29,30]. This is absence in the literature may indicate that NIR-I fluorescent protein has not yet been identified as a suitable BRET acceptor for firefly luciferase.

### 3.3. Development of d-Luciferin Analogs with Longer Wavelengths Based on Molecular Design from Substrate Specificity 

**Figure 1:**
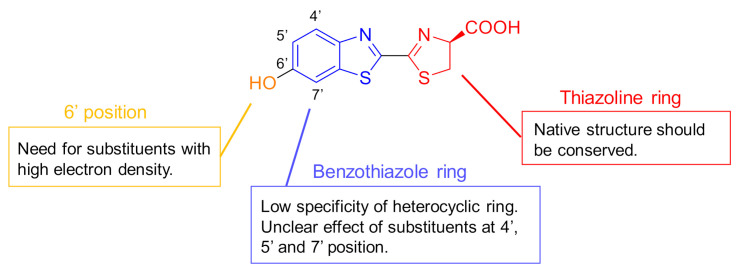
The substrate specificity for luciferase to emit light.

**Figure 2:**
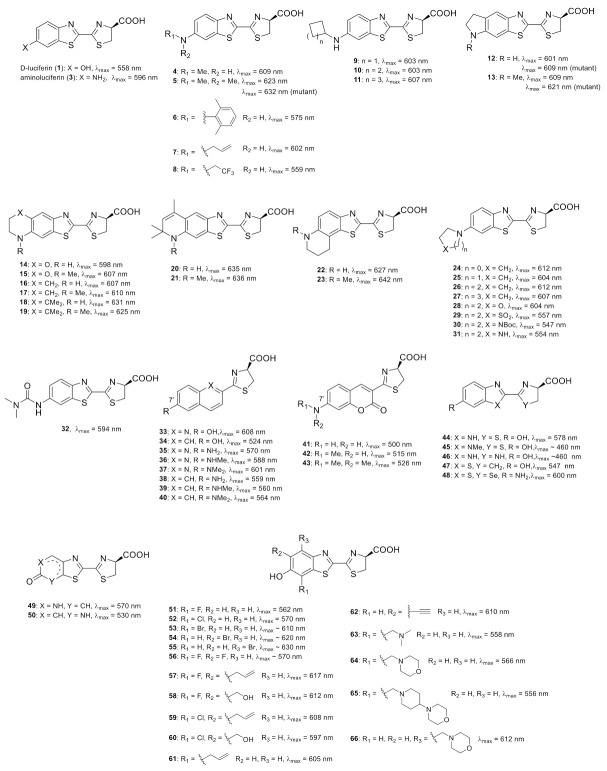
Chemical structures of substrates which were designed using substrate specificity.

Bioluminescent systems with longer luminescence wavelengths have been developed by structurally modifying the substrate. Early studies more than 50 years ago focused on the substrate specificity of d-luciferin for luciferase [31,32,33]. In these studies, it was discovered that the thiazoline ring of the original structure should be conserved for emitting bioluminescence, whereas the aromatic ring and its substituents could be modified, with the requirement that a functional group with high electron density be placed at the 6′ position (Figure 1). These empirical findings became an important design guideline for the development of novel substrates for luciferase. In early studies, aminoluciferin (AL) (Figure 2, Compound **3**), an amino-substituted form of d-luciferin, was reported as a substrate with a longer luminescence peak than d-luciferin; the luminescence spectrum had a maximum peak of 596 nm, was pH-independent, and exhibited a luminescence activity superior to that of other substrates [34]. Since 2000, bioluminescence has been attracting attention as a detection method for assay systems and an imaging method for living animals due to the high sensitivity of the measurement. Consequently, luminescent substrates have been actively developed. Among the studies, the original substrates d-luciferin and AL became important platforms for substrate modification.

First, substrates with modification at the 6′ position were described (Figure 2). As mentioned above, a functional group with high electron density is required at the 6′ position to emit bioluminescence. If the hydroxy group is etherified or esterified, the electron density of the functional group at the 6′ position decreases and no bioluminescence is observed. On the other hand, *N*-alkylation of AL at the 6′ position does not decrease the electron density; therefore, substrates that modified the amino group of AL at the 6′ position have been developed. As in the case of fluorescent dyes containing amino groups, such as rhodamine, alkylation of the amino group of AL was reported to shift the luminescence wavelength up to ~620 nm (Compound **4**, **5**, **7**) [35,36,37]. However, depending on the functional group introduced, the wavelength can be as short as 560 nm, suggesting that other factors are also involved in the luminescence wavelength (Compound **6**, **8**) [37]. AL with cycloalkyl groups showed a slightly longer wavelength shift to ~607 nm than AL (Compound **9**–**11**) [38]. Since it is known that the fluorescence wavelength of rhodamine can be red-shifted by introducing an alkyl groups through a ring fusion rather than alkyl chain groups, substrates based on the same molecular design were also developed (Compound **12**–**23**) [35,39]. Among them, Compound **23** showed the longest luminescence wavelength (642 nm). In addition, substrates with cyclic amino groups were also developed (Compound **24**–**31**) [37]. These substrates showed a long wavelength shift of ~612 nm, which is comparable to that of the substrates with alkyl chain groups (Compound **4**, **5**). Normally, substitution at the 6′ position with an amide group results in loss of bioluminescence activity of AL due to a decrease in electron density at the 6′ position, but the substrate with a dimethylurea group (Compound **32**) exhibited luminescence and showed a luminescence wavelength of 594 nm, which is comparable to AL [37].

Next, studies focusing on the modification of heterocycles and heteroatoms of heterocycles were described (Figure 2). In early studies, bioluminescence was reported using substrates with quinoline and naphthalene rings instead of benzothiazole rings, exhibiting a red shift of 608 nm for a quinoline ring and a blue shift of 524 nm for a naphthalene ring (Compound **33**, **34**) [40]. Quinoline- and naphthalene-containing substrates were also developed with amino groups at the 7′ position, showing luminescence wavelengths of 570–601 nm in the quinoline ring and 559–564 nm in the naphthalene ring (Compound **35**–**40**) [41]. A substrate comprising a coumarin ring with amino groups at the 7′ position showed an even shorter luminescence wavelength from 500 to 526 nm (Compound **41**–**43**) [41]. Further, substrates with a benzimidazole ring instead of the benzothiazole ring or imidazoline ring instead of the thiazole ring were synthesized (Compound **44**–**46**) [42]. Compound **44** showed biphasic luminescence wavelengths with the peak tops at 460 nm and 578 nm, likely due to tautomerism of the emitter. Replacement of the sulfur atom in the thiazoline ring of d-luciferin and AL with carbon or selenium resulted in luminescence wavelengths of 547 nm and 600 nm, respectively (Compound **47**, **48**) [43,44]. Substitution of pyridone ring instead of the benzothiazole ring showed a luminescence wavelength of 570 nm or 530 nm (Compound **49**, **50**) [45].

d-luciferin analogs with varied substituents at the 4′, 5′, and 7′ positions were also developed (Figure 2). The substrates with a halogen at the 4′, 5′, and 7′ positions tended to shift to longer wavelengths than d-luciferin (Compound **51**–**56**) [14,46,47]. Among them, the substrate with bromine at the 4′ position (Compound **55**) showed the longest luminescence wavelength of 630 nm. The substrates with allyl, hydroxymethyl, or alkynyl groups at the 5′ or 7′ positions showed longer luminescence wavelengths up to 617 nm (Compound **57**–**62**) [14,48,49]. When a bulkier functional group was introduced into the 7′ position of d-luciferin, the luminescence wavelengths were almost identical to d-luciferin (Compound **63**–**65**), while the substitution at the 4′ position resulted in a longer wavelength shift to 612 nm (Compound **66**) [50].

### 3.4. Development of d-Luciferin Analogs with Expanded π Conjugation System 

**Figure 3:**
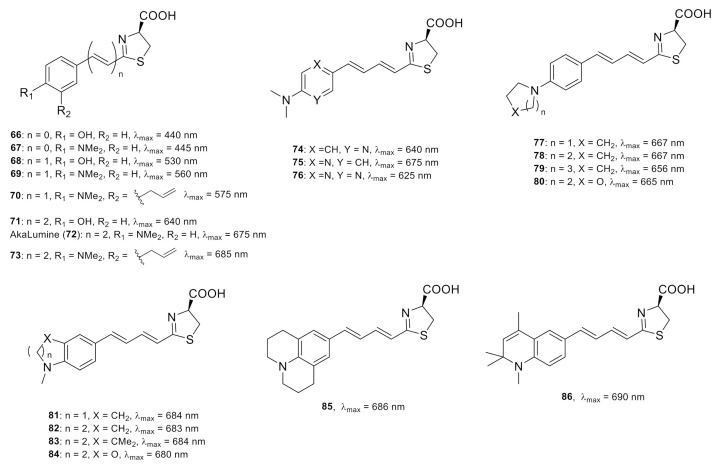

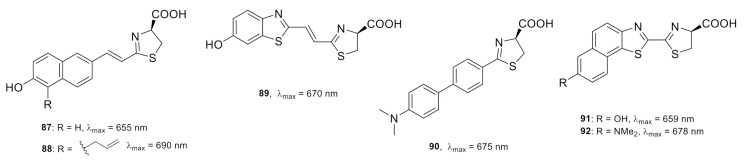
Chemical structures of substrates with elongated π-conjugated systems.

Substrates that were developed based on the initial findings on the substrate specificity of luciferase did not successfully exhibit luminescence peak wavelengths in the optical window. Thus, for the development of NIR-I emitting substrates, a different design strategy was attempted. Maki et al. found that substrates with a double bond inserted between the aromatic ring and the thiazoline ring of d-luciferin and AL also exhibits luminescence, and that this type of luminescent substrate, as in the case of fluorescent dyes, showed a narrowing of the highest occupied molecular orbital (HOMO)-lowest unoccupied molecular orbital (LUMO) gap due to the elongation of the π-conjugated system, leading to a red-shift of the luminescence wavelength. This research resulted in the development of numerous substrates with elongated π-conjugated systems (Figure 3). Maki and co-workers synthesized substrates consisting of a thiazoline ring directly bonded to either a phenol or *N*,*N*-dimethylaniline or connected via a double bond linker (Compound **67**–**70**, **72**, **73**) [51]. In the case of the same heterocyclic structure, substrates with a dimethylamino group showed a longer luminescence wavelength of about 15–35 nm compared to those with a hydroxy group. In addition, the insertion of a double bond between the two hetrocyclic ring structures led to a longer wavelength shift of about 100 nm per bond. In particular, Compound **73**, named AkaLumine, was found to be a substrate with a maximum luminescence wavelength in the optical window. After that, based on the structure of AkaLumine, various substrates were developed. The introduction of an allyl group at the 7′ position in AkaLumine was expected to cause a long wavelength shift, as in the case of d-luciferin (Compound **71**, **74**) [52]. As a result, a longer wavelength shift of about 10–15 nm was observed compared to the unsubstituted substrates. AkaLumine did not exhibit good solubility in water, so a more water-soluble substrate was developed by introducing a nitrogen atom into the benzene ring (Compound **75**–**77**) [53]. The luminescence wavelength changed depending on the position of nitrogen (620–675 nm). Since the luminescence wavelength of AL was changed by the introduction of a cyclic amine groups and ring-fused alkyl amino groups, the same molecular design was applied to AkaLumine (Compound **78**–**87**) [54,55]. Among the developed substrates, Compound **87** had the longest luminescence of 690 nm. In addition to phenol and *N*,*N*-dimethylaniline, additional substrates were prepared bearing other heterocyclic rings, including naphthalene rings and benzothiazole rings connected via double bond linkers (Compound **88**–**90**) [51,52,56]. All of these substrates were shifted to longer wavelengths by about 100 nm compared to the analogous substrates lacking the double bond linker.

**Figure 4:**
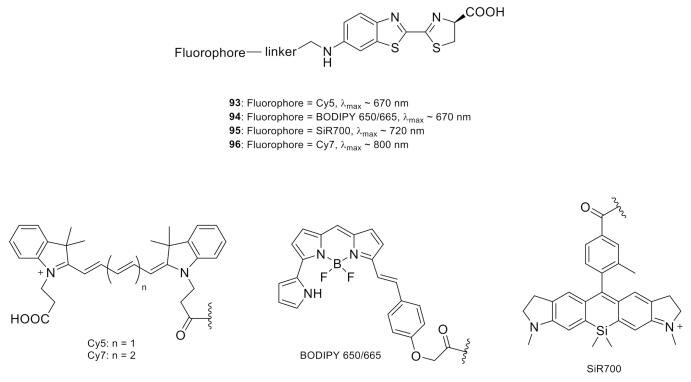
Chemical structures of substrates based on bioluminescence resonance energy transfer (BRET).

Other substrates with heterocyclic rings, such as biphenyl and naphtho[2,1]thiazole rings, were developed (Compound **91**–**93**) [57,58]. All of these substrates showed luminescence wavelengths in the NIR-I region of 659–678 nm. Substrates with longer luminescence wavelengths utilizing bioluminescence resonance energy transfer (BRET) were also reported (Compound **94**–**97**) (Figure 4) [59]. Fluorescent dyes with a wavelength in the NIR-I region, such as Cy5, BODIPY 650/665, SiR700, Cy7, etc., were attached to the amino group of AL via a linker. In these substrates, BRET from the AL to the NIR-I fluorescent dye efficiently occurred and showed luminescence wavelengths in the NIR-I region.

### 3.5. Mutant of Luciferase for Synthetic Substrates

Although various types of NIR-I light-emitting substrates for luciferase have been developed, almost none of them emit brighter light than d-luciferin; indeed, many of them emit less than 1%, and in the worst case, some of them emit less than 0.01% [51]. The main reason for this is thought to be that the adenylation reaction, the first step in the bioluminescence reaction, is very slow. For example, a substrate with a pyrroline ring instead of a thiazole ring (Compound **47**) shows only about 0.5% luminescence compared to d-luciferin. However, when the adenylated substrate reacted with luciferase, the luminescence intensity was drastically improved to about 23% compared to adenylated d-luciferin [44]. Unfortunately, adenylated substrates could not be used as substrates because they do not readily penetrate the cell membrane due to the negatively charged phosphate group, which renders them unstable. Therefore, it would be advantageous to instead increase the reaction rate of the adenylation step, thereby improving the light output. To that end, research has been conducted to find luciferases with optimal active sites for specific substrates through the large-scale screening of mutation. The first attempt was to screen for optimal luciferases for AL derivatives **12** and **13**; as a result, mutants for Compound **12** and **13** with improved luminescence were found [60]. In several similar studies, mutants showing longer wavelengths also have been discovered. For example, mutants for Compound **90** and **92** emit red-shifted bioluminescence with peak wavelengths from 670 nm to 706 nm and 659 nm to 758 nm, respectively, compared to the native luciferase [56,58]. The most successful example of the mutation studies is probably Akaluc against Akalumine, reported by Miyawaki et al. Akaluc has mutations of 28 amino acids and is more than 1000 times brighter than the native luciferase [61]. Using the system with AkaLumine and Akaluc, Miyawaki et al. observed luminescence from a single cell in a live mouse and a brain of freely moving mouse. They also successfully imaged bioluminescence from the brain of a much larger animal, the marmoset. This system is one of the most sensitive luminescence systems available today for in vivo imaging.

## 4. Bioluminescence Probe

### 4.1. Strategy for the Development of Bioluminescence Probes

**Figure 5:**
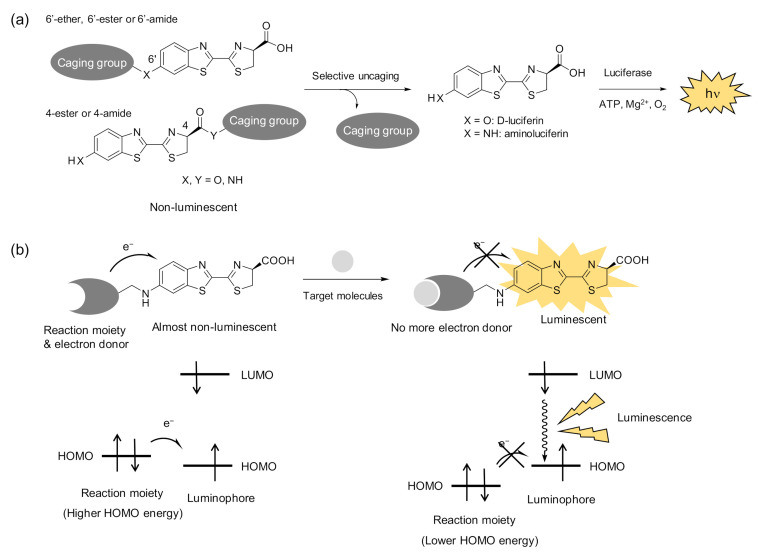
Molecular designs for the development of bioluminescence probes. The design strategy of (**a**) caged luciferin and (**b**) bioluminescent-enzyme induced electron transfer (BioLeT). The rate of electron transfer is strongly dependent on the highest occupied molecular orbital (HOMO) energy level of the electron donor. If the HOMO energy level of the electron donor is enough high, after luciferin derivatives react with luciferase, the electron transfer easily occurs from the electron donor to the excited luminophore. In this case, the excited electron cannot return to the HOMO of the luminophore, resulting in loss of bioluminescence. When the HOMO energy level of the electron donor becomes lower after reaction with target molecules, the electron transfer does not occur anymore, and bioluminescence should be restored.

The substrates described above are mainly used for the imaging of luciferase as a reporter enzyme, for example, to monitor gene expression, promoter activity, and cell tracking. On the other hand, bioluminescence probes, so-called bioluminogenic probes or activatable probes, have been developed to detect specific molecules or enzyme activities by modifying the substrate structure. Bioluminescence probes do not emit light when reacting with luciferase or do not function as a luminescent substrate. However, when the bioluminescence probe reacts with the target molecules, the probe changes its structure and becomes a luminescent substrate, finally emitting light in the presence of luciferase. Two rational strategies for the molecular design of bioluminescence probes have been utilized so far: one is the caged luciferin strategy and the other is the strategy utilizing bioluminescence quenching by electron transfer in the excited state (Figure 5). The caged luciferin strategy focuses on the functional groups of the substrate required for bioluminescence. Bioluminescent activity is initially masked by a reaction moiety of the target molecule; but reaction with the target molecule causes deprotection of the reaction site and subsequent release or generation of d-luciferin or AL, resulting in bioluminescence. For example, the 6′-*O*-ether and 6′-*O*-ester derivatives of d-luciferin are known to be non-luminescent substrates and are actually used as inhibitors. Therefore, when a reactive moiety for the target molecule is attached to d-luciferin at the 6′ position via an oxygen atom, this substrate does not emit light. However, when the target molecule reacts with the reactive moiety, the substrate is converted to d-luciferin and light is emitted. Using this strategy, it is possible to detect and quantify the target molecule by luminescence. Similarly, since amidation of AL at the 6′ position results in the loss of its bioluminescent activity, the 6′-*N*-amide has been used in the reaction site for the molecular design of caged luciferin. Another masking site is the carboxylic acid at position 4 of the thiazoline ring. Adenylation of the carboxylic acid is essential for the bioluminescence reaction, but in amide and ester forms, the adenylation reaction does not proceed anymore and the luminescence activity is lost. Therefore, 4-ester and 4-amide derivatives of d-luciferin have been used for caged luciferin-type bioluminescence probes. These molecular designs can be applied to target molecules that cause a cleavage reaction for a specific structure. Using the caged luciferin strategy, many bioluminescence probes, such as detecting enzyme activity, small molecules, and metals, have been developed so far, as will be shown in the next section. The other strategy for developing bioluminescence probes relies upon photoinduced electron transfer (PeT), which has been used previously to develop fluorescence probes [62,63,64]: Bioluminescence can be quenched by the electron transfer from the donor moiety in the vicinity to the substrate (luminophore) in the excited state, although the excitation process is different (i.e., by excitation light for fluorescence and chemical reaction for bioluminescence). This phenomenon is called bioluminescent-enzyme induced electron transfer (BioLeT) [65]. The electron transfer is controlled by the electron density of the electron donor moiety, so the bioluminescence can be also controlled by changing the electron density. The strategy of using caged luciferin is applied to target molecules that cause cleavage reactions, while BioLeT is applied to target molecules that change the electron density of the reaction moiety.

### 4.2. Development of Bioluminescence Probes Based on a Caged Luciferin Strategy

**Figure 6:**
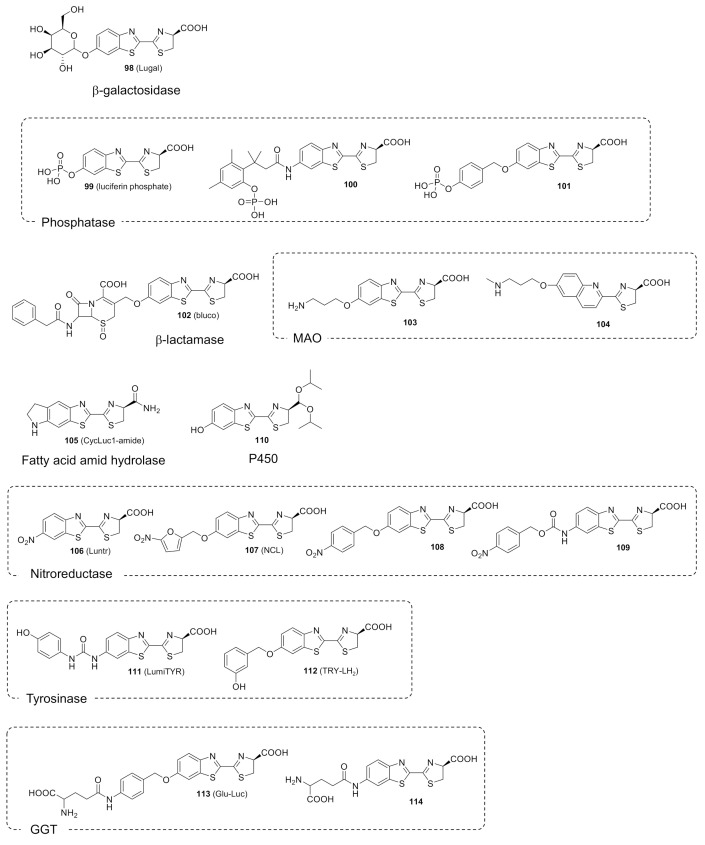

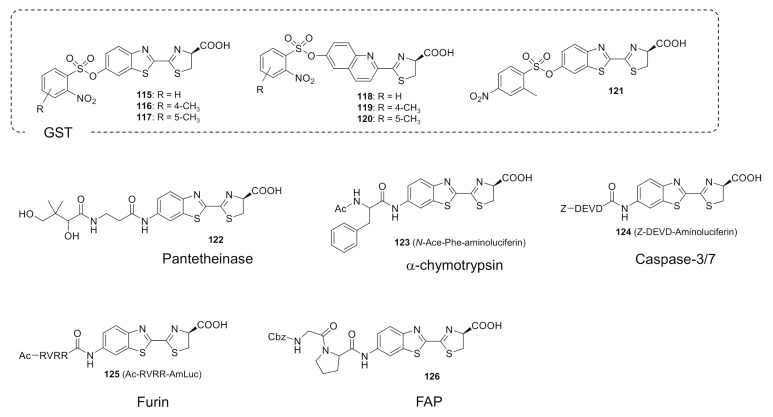
Chemical structures of bioluminescence probes for the detection of enzyme activity.

**Figure 7:**
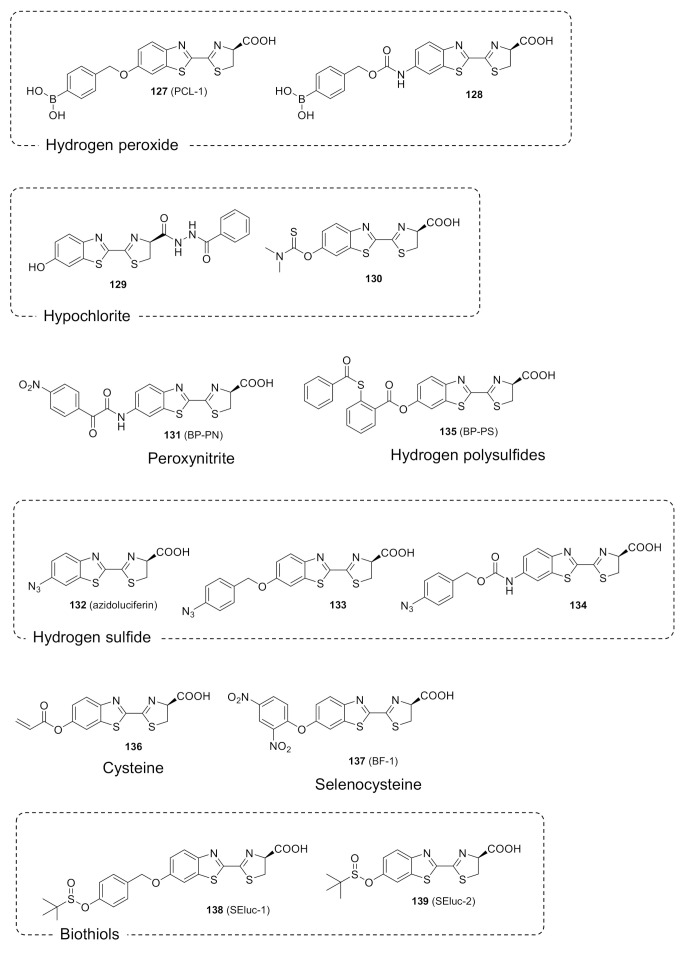
Chemical structures of bioluminescence probes for small molecules.

**Figure 8:**
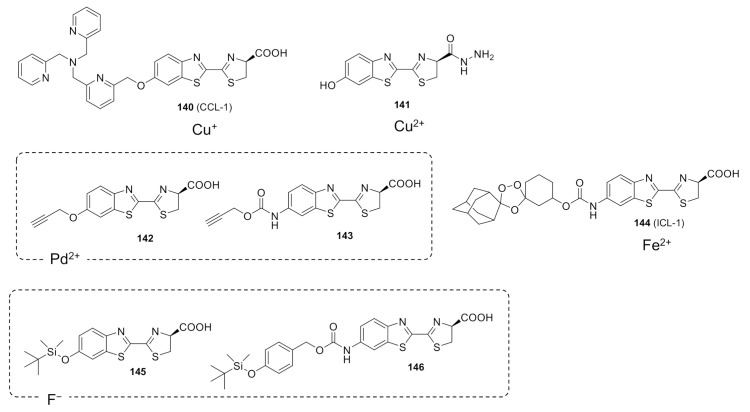
Chemical structures of bioluminescence probes for ions.

**Figure 9:**
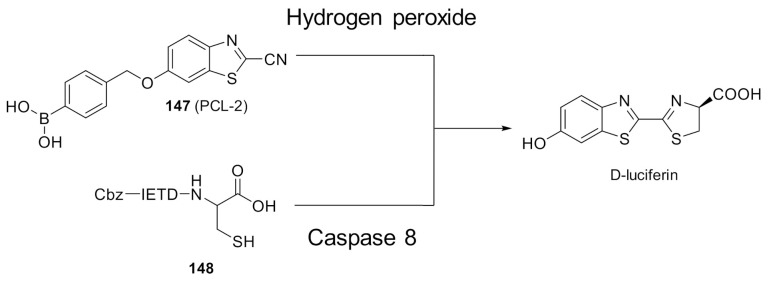
Chemical structures of bioluminescence probe for dual analytes.

The first bioluminescence probe was developed to detect the activity of β-galactosidase using a caged luciferin (Compound **98**) [66]. A β-galactosyl group was attached to d-luciferin at the 6′ position, resulting in loss of luminescence activity. However, in the presence of β-galactosidase, the galactosyl group is cleaved to produce d-luciferin, and the enzyme activity can be detected by bioluminescence. The strategy of using a caged luciferin has been employed to develop many probes for detecting enzyme activity (Figure 6). For example, detection of phosphatase (Compound **99–101**) [67,68], β-lactamase (Compound **102**) [69], monoamine oxidase (MAO) (Compound **103**, **104**) [70], fatty acid amide hydrolase (Compound **105**) [71], nitroreductase (Compound **106–109**) [72,73,74,75], P450 (Compound **110**) [76], tyrosinase (Compound **111**, **112**) [77,78], γ-glutamyl transferase (Compound **113**, **114**) [79,80], glutathione S-transferase (GST) (Compound **115–121**) [81], and pantetheinase (Compound **122**) [82], etc., are all based primarily on the 6 ‘-*O*-ether type of d-luciferin or the 6′-*N*-amide type of AL. In addition, the 6′-*N*-amide type of AL has been used for detecting endopeptidases, such as, α-chymotrypsin (Compound **123**) [83], caspase 3/7 (Compound **124**) [84], furin (Compound **125**) [85], and fibroblast activation protein-α (Compound **126**) [86]. In addition to enzymes, other targeted biomolecules include reactive oxygen species, such as hydrogen peroxide (Compound **127**, **128**) [87,88], hypochlorite (Compound **129**, **130**) [89,90], and peroxynitrite (Compound **131**) [91], active sulfur molecular species, such as hydrogen sulfide (Compound **132–134**) [92,93] and polysulfide (Compound **135**) [94], and biothiols (Compound **136–139**) [95,96,97] (Figure 7). Further, bioluminescence probes for ions, such as copper (Compound **140**, **141**) [98,99], palladium (Compound **142**, **143**) [100], iron (Compound **144**) [101], and fluorine (Compound **145**, **146**) [102], have also been reported (Figure 8). Bioluminescence probes have also been developed for dual target molecules (Compound **147**, **148**) [103] (Figure 9): For example, Compound **147** and **148** react with hydrogen peroxide and caspase 8 to release a luciferin precursor and d-cysteine, respectively. Since the two molecules react with each other under physiological conditions to produce d-luciferin, luminescence is generated only when both hydrogen peroxide and caspase8 are present. These various examples illustrate that bioluminescence probes have been successfully used for a variety of target molecules using the caged luciferin strategy.

**Figure 10:**
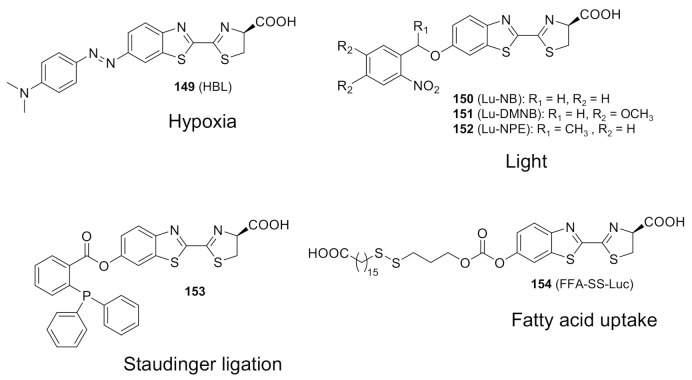
Chemical structures of bioluminescence probes for hypoxia, light, Staudinger ligation, and fatty acid uptake.

The caged luciferin strategy has been applied not only to the detection of biomolecules but also the environment, external stimuli, chemical reactions and biological events (Figure 10). For example, the bioluminescence probes have been reported whose structures are altered by hypoxia in the cell (Compound **149**) [104] or by light (Compound **150**–**152**) [105]. In addition, bioluminescence probes can respond to the Staudinger ligation (Compound **153**) [106]. In Staudinger ligation, a trivalent phosphorus compound reacts with an azide compound to give an iminophosphorane compound, which then undergoes hydrolysis. Here, the hydrolysis reaction is designed to produce d-luciferin as a hydrolysis product. By applying the probe to cells that are taken up azide sugars, it is possible to monitor the glycome on the surface of the cell membrane which changes according to the developmental stage, cellular activation, and so on. Another bioluminescence probe that reacts with intracellular thiols to produce d-luciferin when taken up into the cell has also been developed (Compound **154**) [107]. By attaching the fatty acid to the probe, the amount of fatty acid uptake can be quantified.

In the development of caged luciferin-type bioluminescence probes, it is necessary to consider whether the reaction moieties should be attached to the substrates directly or through a linker, and whether a 6′-*O*-ether type of d-luciferin or 6′-*N*-amide type of AL is better. The performance of bioluminescence probes with the different structures for the same target molecules have been compared in several papers. In the probes detecting the activity of nitroreductase, a nitro group functions as the reactive moiety. One study reported the development of three probes; the direct conjugate type (Compound **106**), d-luciferin benzylether linker type (Compound **108**), and AL benzyloxycarbonyl linker type (Compound **109**) [75]. In a comparison of these three probes, Compound **108** showed the best performance as a nitroreductase probe, perhaps because of enzyme recognition. In the case of the hydrogen sulfide probe, an azido group was used as the reactive moiety. Like nitroreductase probes, the direct conjugate type (Compound **132**), d-luciferin benzylether linker type (Compound **133**), and AL benzyloxycarbonyl linker type (Compound **134**) were compared and Compound **132** had the best reactivity for hydrogen sulfide [93]. In the biothiol probes which contained a tert-butyl sulfinyl group as the reaction moiety, the d-luciferin benzylether type (Compound **138**) was more reactive than the direct conjugate type (Compound **137**) [97]. In the development of palladium ion probes with a propargyl group as the reaction moiety, the AL oxycarbonyl linker type (Compound **143**) was better than the d-luciferin ether type (Compound **142**) [100]. In the fluorine ion probes whose reaction moiety is tert-butyl dimethylsilyl group, the direct conjugate type (Compound **145**) was superior to an AL benzyloxycarbonyl linker (Compound **146**) [102]. Thus, the optimum structure for a particular target molecule depends upon both enzyme recognition and the reactivity; therefore, it is best to optimize the reaction moiety when developing bioluminescence probes.

### 4.3. Development of Bioluminescence Probes Based on the BioLeT Strategy

**Figure 11:**
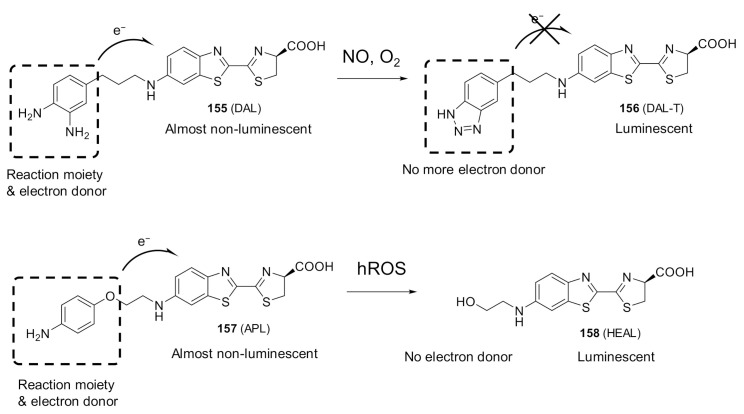
Chemical structures of bioluminescence probes based on bioluminescent-enzyme induced electron transfer (BioLeT).

Next, the development of a bioluminescence probe using BioLeT was described. As mentioned earlier, the basic concept of BioLeT is the same as that of PeT. In other words, bioluminescence can be quenched in the excited state by electron transfer from the electron donor to the emitter, although the excitation process is different. The molecular design of caged luciferin can be applied to the target molecules involved in the reaction of bond breaking, while BioLeT can target chemical reactions that cause changes in electron density (HOMO energy level). One advantage of probes based on BioLeT is that the chemical reactions in which the target molecule binds or coordinates to the reaction moiety can be applied to the molecular design of the probe, as long as the electron density of the reaction moiety changes before and after the reaction. So far, bioluminescence probes utilizing BioLeT for the detection of nitric oxide (Compound **155**) and highly reactive oxygen species (hROS) (Compound **157**), such as hypochlorite, peroxynitrite, and hydroxyl radical, have been developed (Figure 11) [65,108]. Compound **155** has a diaminobenzene structure whose electron density is very high, so even in the presence of luciferase, electron transfer from the diaminobenzene to the emitter occurs, resulting in loss of bioluminescence. The structure is also the reaction moiety for nitric oxide as the target molecule, and, when reacted with nitric oxide in the presence of oxygen, it is converted to a triazole structure (Compound **156**). The electron density of the structure is reduced, so that electron transfer from the triazole to the emitter does not occur, thus recovering luminescence. Similarly, Compound **157** bearing an aminophenoxy moiety as a reaction moiety does not exhibit bioluminescence in the presence of luciferase because the electron density of the aminophenoxy moiety is sufficient to quench bioluminescence. The aminophenoxy moiety can react with hROS and is cleaved via an ipso-substitution reaction. Therefore, after the reaction, Compound **157** is converted to Compound **158**, which has no reaction moiety with high electron density, and shows bioluminescence. Although there are only two examples of bioluminescence probes using BioLeT, it is expected that bioluminescence probes based on this design principle will be developed in the future because the same molecular design as PeT can be applied.

## 5. Chemiluminescence

**Figure 12:**
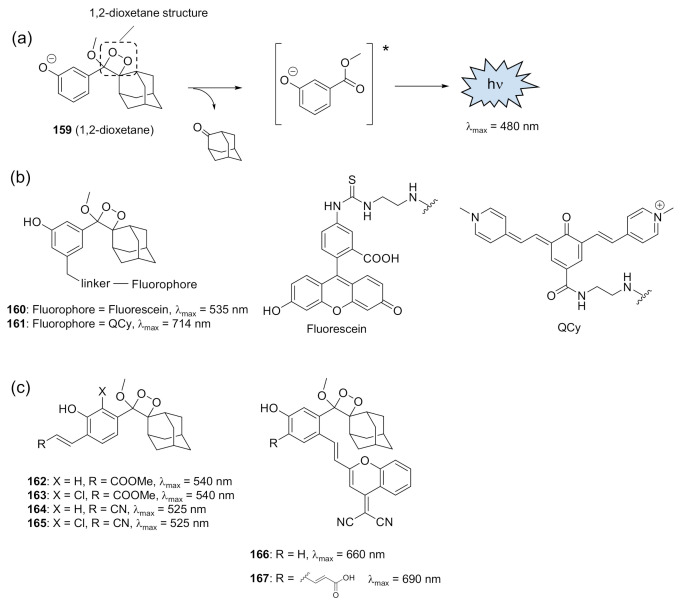
(**a**) Chemiluminescence reaction of 1,2-dioxetane. (**b**) Chemical structures of chemiluminescent compounds conjugated with fluorescent dyes. (**c**) Chemical structures of chemiluminescent compounds with high brightness.

**Figure 13:**
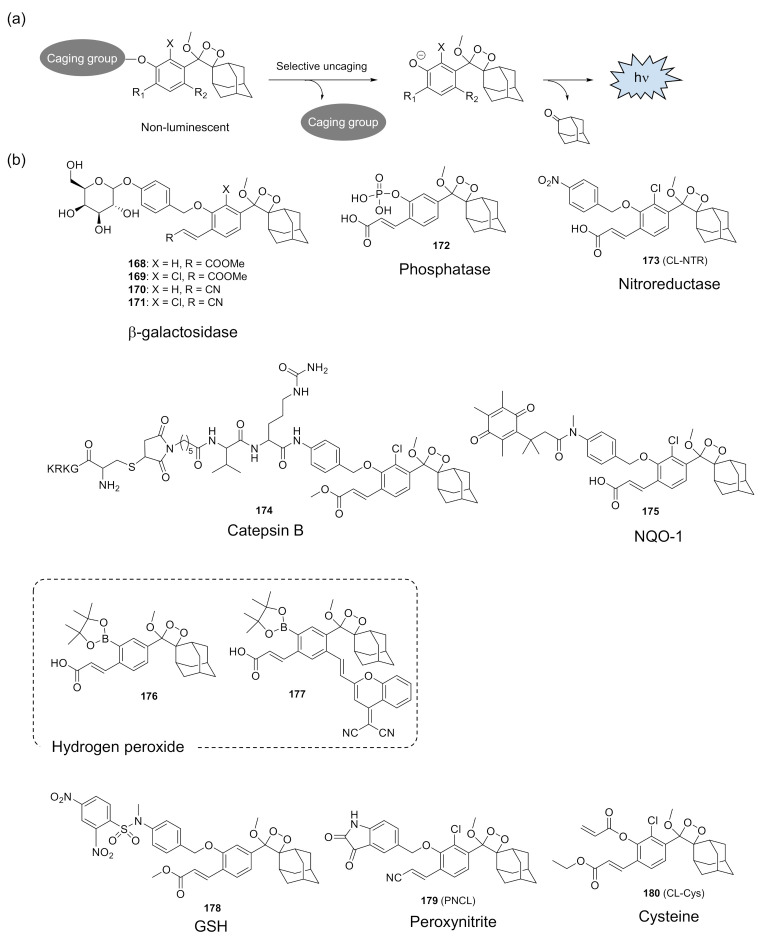

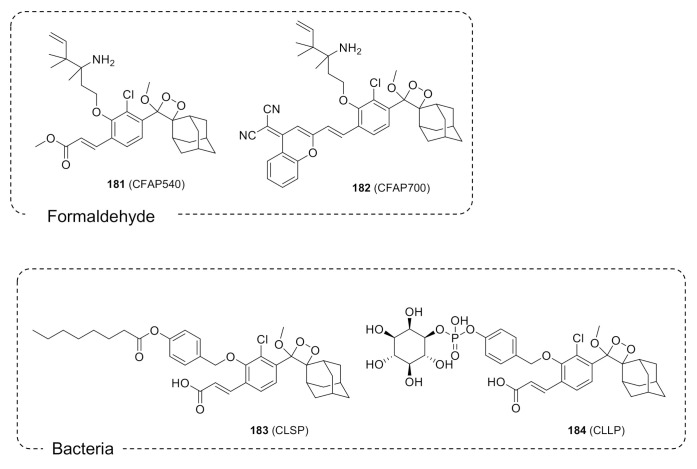
Chemiluminescence probes based on the caged strategy. (**a**) The design strategy of caged compounds. (**b**) Chemical structures of chemiluminescence probes.

Chemiluminescence, which is produced by chemical reaction in absent of enzyme/protein, is a well-known phenomenon. One of the most famous chemiluminescent compounds is luminol, which reacts with reactive oxygen species, followed by the generation of the high-energy four-membered ring peroxide, so-called 1,2-dioxetane. Since the O–O bond has enough energy to allow the molecule to decompose thermally into an electronically excited product, the 1,2-dioxetane compound is spontaneously excited with decomposition into two carbonyl fragments, resulting in production of light. So far, chemiluminescence has been applied to detection systems in biochemical and bioimaging research [109,110]. However, chemiluminescence has not attracted as much attention as bioluminescence because the brightness in neutral aqueous solution is not high and the luminescence wavelength is limited due to a decreased variation in chemiluminescent compounds. Recently, Shabat et al. developed chemiluminescent compounds with high brightness in neutral aqueous solution. They used 1,2-dioxetane stabilized with an adamantane structure as a basic structure of the synthesized chemiluminescent compounds (Compound **159**) (Figure 12). The compounds possess a phenol group, which upon conversion to the phenolate in the decomposition reaction is excited, resulting in generation of chemiluminescence. Shabat and co-workers developed a series of very bright chemiluminescent compounds by attaching fluorescent dyes with high fluorescence quantum yields utilizing chemiluminescence resonance energy transfer (CRET) (Compound **160**, **161**) (Figure 12) [111]. This molecular design worked well, and the excitation energy before relaxation was efficiently transferred to the fluorescent dyes, resulting in bright chemiluminescence. However, they found that the compounds based on the concept of CRET were unstable to light due to electron transfer from the fluorescent dyes absorbing light. To develop other chemiluminescent compounds with high brightness, they focused on the chemiluminescence quantum yields which consist of the reaction quantum yield, singlet excitation quantum yield and fluorescence quantum yield of the emitter. To improve the fluorescence quantum yield of the emitter, they synthesized 1,2-dioxetanes containing an acrylic group (Compound **162**–**165**) (Figure 12) [112]. With this approach, they successfully developed chemiluminescent compounds with high brightness in aqueous solution. In recent years, there has been a great deal of research using such compounds. For example, chemiluminescent compounds with longer luminescence wavelengths were prepared by extending the π-conjugated system (Compound **166**, **167**), the half-life time of the 1,2-dioxetane compounds, which depends on structures (2–1100 s), was investigated, and the chemiluminescence probes have been synthesized [113,114]. The chemiluminescence reaction of 1,2-dioxetanes proceeds from its phenolate form, and this reaction can be inhibited by introducing a protecting group at the site. Therefore, the strategy of caged luciferin can be applied to the development of chemiluminescence probes. Although the chemiluminescent compounds have only been developed for a short time, to date various chemiluminescence probes have been reported, including chemiluminescence probes for detecting the activities of enzymes, such as β-galactosidase (Compound **168**–**171**) [112,115], phosphatase (Compound **172**) [112], nitroreductase (Compound **173**) [116], cathepsin B (Compound **174**) [117], and nicotinamide adenine dinucleotide/nicotinamide adenine dinucleotide phosphate (NADH/NADPH) quinone oxidoreductase-1 (NQO1) (Compound **175**) (Figure 13) [118]. In addition, chemiluminescence probes have been designed to detect small molecules, such as hydrogen peroxide (Compound **176**, **177**) [112,113], glutathione (GSH) (Compound **178**) [112], peroxynitrite (Compound **179**) [119], cysteine (Compound **180**) [120], and formaldehyde (Compound **181**, **182**) (Figure 13) [121]. Further, chemiluminescence probes for bacteria have been developed utilizing reaction moieties for specific bacterial enzymes (Compound **183**, **184**) (Figure 13) [122]. Several probes have been shown to be more sensitive than fluorescence probes and have been applied to in vivo imaging. Various studies are expected to be conducted in the future.

While chemiluminescence and bioluminescence show almost similar sensitivities, bioluminescence requires luciferase for measurement, which has both advantages and disadvantages. For experiments investigating gene expression or cell tracking, a reporter enzyme is required. In such a case, bioluminescence is more suitable because luciferase can be used directly as a reporter enzyme. In principle, no signal is detected from cells that do not express luciferase, so a highly sensitive measurement is possible. On the other hand, in the case of the development of imaging probes for biomolecules, luciferase is required for the bioluminescence measurement, which then requires the preparation of transfected cells and transgenic animals expressing luciferase, leading to less flexibility and higher costs. The chemiluminescence probes do not have such problems, thus allowing us to establish a simpler experimental system, which may become an important alternative imaging tool in the future.

## 6. Summary

Since luminescence imaging has a lower background signal compared to fluorescence imaging, it will continue to be used, especially for in in vivo imaging. As outlined in this review, various studies on bioluminescence imaging reagents have been conducted in the last two decades, and there has been a great deal of interest in developing substrates for in vivo imaging and bioluminescence probes for detecting biomolecules and in vivo events. In addition, a very large number of caged luciferin-based bioluminescence probes have been developed and applied to in vivo imaging. On the other hand, chemiluminescence probes based on the 1,2-dioxetane scaffold with higher brightness have also been rapidly developed and have applied to in vivo imaging. It is expected that bioluminescence and chemiluminescence imaging will continue to be used as powerful analytical tools, and that new biological findings will be discovered from these methods.
